# Expression of inflammatory mediators in biofilm samples and clinical association in inflammatory bowel disease patients—a preliminary study

**DOI:** 10.1007/s00784-021-04093-2

**Published:** 2021-08-12

**Authors:** Mayte Buchbender, Jakob Fehlhofer, Peter Proff, Tobias Möst, Jutta Ries, Matthias Hannig, Markus F. Neurath, Madline Gund, Raja Atreya, Marco Kesting

**Affiliations:** 1 Department of Oral and Maxillofacial Surgery, University of Erlangen- Nuremberg, Glückstrasse 11, 91054 Erlangen, Germany; 2 Head of the Department of Orthodontics, University of Regensburg, Regensburg, Germany; 3 Head of Department of Clinic of Operative Dentistry, Periodontology and Preventive Dentistry, Saarland University, Homburg, Germany; 4 Department of Medicine, University of Erlangen-Nuremberg, Erlangen, Germany; 5 Deutsches Zentrum Immuntherapie DZI, University of Erlangen-Nuremberg, Erlangen, Germany; 6 Department of Clinic of Operative Dentistry, Periodontology and Preventive Dentistry, Saarland University, Homburg, Germany

**Keywords:** Crohn’s disease, Ulcerative colitis, Cytokine expression, Oral biofilm, Periodontitis

## Abstract

**Objectives:**

Inflammatory bowel disease (IBD) has multiple impacts on soft and hard tissues in the oral cavity. The aim of this study was to analyze the expression of cytokines in biofilm samples from patients suffering from IBD and compare them to healthy patients. It was hypothesized that different cytokine expression levels and clinical associations might be drawn.

**Material and methods:**

A total of 56 biofilm samples from three different patient cohorts (group 0 = healthy, HC *n* = 30; group 1 = Crohn’s disease, CD, *n* = 19; group 2 = ulcerative colitis, UC, *n* = 7) were examined for the expression levels of the cytokine interleukins IL-2, -6, and -10; matrix metalloproteinases 7 and 9; and surface antigens CD90/CD11a by quantitative real-time PCR and according to clinical parameters (plaque index, BOP, PD, DMFT, CAL). Relative gene expression was determined using the ∆∆CT method.

**Results:**

The mean BOP values (*p* = 0.001) and PD (*p* = 0.000) were significantly higher in the CD group compared to controls. Expression of IL-10 was significantly higher in the CD (*p* = 0.004) and UC groups (*p* = 0.022). Expression of MMP-7 was significantly higher in the CD group (*p* = 0.032). IBD patients treated with TNF inhibitors (*p* = 0.007) or other immunosuppressants (*p* = 0.014) showed significant overexpression of IL-10 compared to controls.

**Conclusion:**

Different expression levels of IL-10 and MMP-7 were detected in plaque samples from IBD patients. As only BOP was significantly increased, we conclude that no clinical impairment of periodontal tissue occurred in IBD patients.

**Clinical relevance:**

With the worldwide increasing incidence of IBD, it is important to obtain insights into the effects of the disease on the oral cavity. The study was registered (01.09.2020) at the German clinical trial registry (DRKS00022956).

**Clinical trial registration:**

The study is registered at the German clinical trial registry (DRKS00022956).

## Introduction

Inflammatory bowel diseases (IBD) include two major subtypes: Crohn’s disease (CD) and ulcerative colitis (UC), which can affect the entire gastrointestinal tract. These types of chronic IBD are diagnosed by correlating clinical, radiological, endoscopic and histopathological findings and present a chronically relapsing or remitting course (1, 2). The highest age-specific incidence of IBD occurs in the second to third decade of life and is reported to be approximately 100–200 per 100,000 for UC and 6.6 per 100,000 for CD (3, 4).

The etiology of IBD is multifactorial and triggered by many factors, with immunological dysfunction ultimately underlying its development (5, 6). The literature currently discusses the influence of the local microbiological gut flora and the influence of environmental factors, like nicotine consumption, which can cause serious deterioration in disease symptoms those with CD but has been shown to have a protective effect on clinical activity in those with UC (7–9). Changes in the oral mucosa (e.g., fistulas, stomatitis, cobblestone mucosa) as a specific (direct consequence of IBD) or non-specific consequence (e.g., malnutrition or malabsorption) and changes in the periodontium can also be observed (10, 11).

Periodontitis (P) is defined as a “dysbiotic inflammatory disease” in which an imbalance between pathogenic and protective bacteria causes dysregulation of the immune response, leading to an excessive inflammatory reaction and thus periodontitis (12).

Accordingly, clinical studies have shown a correlation between the increased incidence of periodontitis and IBD. A systematic review including 9 studies with a total of 1297 patients showed that CD patients had a higher incidence of pocket depth (PD > 3 mm) and a higher DMFT (decayed-missing-filled-teeth) index (4). Another study showed no association between periodontitis and IBD in 16 UC and 46 CD patients according to the parameters (bleeding on probing (BOP), PD, clinical attachment loss (CAL) and plaque index (PI)). However, overall, plaque accumulation and the incidence of oral lesions were increased in the patients (13).

Another study included a healthy control group (*n* = 74) and found a correlation between periodontitis and IBD according to the parameters CAL, PD and PI; the authors concluded that there was increased periodontitis prevalence in UC patients (*n* = 80) compared to that in CD (*n* = 99) patients (14). The question is how the clinically observed elevated parameters are related to the inflammatory mediators responsible for them in IBD patients.

In CD, the Th1 cell-associated cytokines IFN-γ and IL-2 are more expressed than in UC and healthy controls, while mucosal cells in UC have been shown to produce the Th2-type cytokines IL-5 and IL-13 (10–13). Several studies have shown that there is increased production of Th17 cell-associated cytokines, such as IL-17A and IL-17F, by mucosal T cells in both CD and UC. Furthermore, previous data has shown that there is a correlation between the oral presence of cytokines such as IL-6 and IL-17 in extra-intestinal manifestations in UC or active CD or IL-12 and TNF in extra-intestinal manifestations in CD or in complicated UC (14–17).

In the oral cavity the immune response to pathogens is also triggered by various immune cells and humoural mediators, such as cytokines (15). Cytokine expression in the gingival complex has already been studied, and an increase in pro-inflammatory cytokines such as IL-1ß, -2, -6, -8, tumor necrosis factor (TNF), and interferon-gamma (IFN-γ) has been shown (15–17).

Elevated cytokine levels of IL-6 and TNF in saliva have been shown to be correlated with oral mucosal changes in CD patients and to be equally elevated in periodontitis patients, so it is believed that there are correlations between mucosal changes and the severity and degree of periodontitis based on this evidence, which could also be due to common genetic background between these two entities (4, 18–20).

After the gut microbiome, the oral cavity has the second largest microbiome presence (21–23). Because of the large microbiome and anatomy of the oral cavity, a broad microbial complexity can be found in meta-niches, such as saliva, the palate, the tongue or hard tissue samples of supra-/subgingival plaque (24). In a previous study, higher levels of anti- and pro-inflammatory cytokines in supra-and subgingival plaque sample (granulocyte–macrophage colony-stimulating factor GM-CSF, IFN-γ, IL-4, -6, -8, -10) were found in healthy patients, where niches such as saliva, the tongue, the hard palate, the cheek, and the sublingual area were compared (24).

However, the expression of inflammatory markers within the oral cavity and correlation to clinical parameters in IBD patients remains unclear. Hence, we hypothesize that IBD patients have different cytokine expression compared to that in healthy individuals. Thus, in this study, the expression of cytokines in biofilm samples of patients with UC or CD was clinically examined and compared to that in healthy controls.

## Material and methods

### Study design and patient selection

This observational prospective cohort study reports patients with either ulcerative colitis or Crohn’s disease in comparison to healthy control patients. The patients included in this study presented with either of these diseases and the healthy ones randomly and seeking for dental treatment to the Department of Oral and Maxillofacial Surgery, University of Erlangen-Nuremberg, from March 2019 to December 2020. The diagnosis of CD or UC was made by the Medical Department 1 for gastroenterology of the University Hospital Erlangen from histopathological, radiological, clinical and endoscopic results. Patients were referred to the oral and maxillofacial surgery department during their follow-up after assessment of the current disease activity (as described below).

Ethical approval (no.: 399_18 B) for this study was obtained from the ethics committee of the medical faculty of Friedrich-Alexander University Erlangen-Nuremberg, Germany, and the guidelines of the Declaration Helsinki were followed. The study was also registered in the German clinical trial registry (DRKS00022956). After obtaining signed informed consent, patients of all ages and of both sexes were included. The included patients were examined for one hour during one visit and were assigned to group 0 (healthy control, HC), group 1 (patients suffering of Crohn’s disease, CD) and group 2 (patients suffering of ulcerative colitis, UC) according to their underlying disease, respective missing disease.

The exclusion criteria for all patients of groups 0, 1, and 2 were:

- Smoking/nicotine abuse
- Diabetes type I and II
- Already diagnosed with periodontitis or had already undergone periodontitis treatment (ever)
- Poor general health that did not permit a detailed dental examination
- Toothlessness
- Prior antibiotic treatment (neither systemic nor local administration at least 4 weeks)

### Clinical examination and sampling procedures

All the sampling procedures and clinical parameters were performed and assessed by one dentist (J.F) according to a standardized procedure during the initial presentation of patients to the Department of Oral and Maxillofacial Surgery.

The following clinical parameters were collected:

- Mombelli Plaque Index (PI), graded from 0 to 3 (grade 0: no plaque detected by inspection or probing; grade 1: accumulation of plaque was visible only by probing the sulcus with a probe but not by eye; grade 2: visible plaque accumulation; grade 3: massive plaque accumulation)
- Bleeding on probing (BOP) in %
- Pocket depth (PD—distance from the marginal gingival margin to the bottom of the pocket) on 6 sites (mesio-buccal, buccal, disto-buccal, mesio-lingual, lingual, disto-lingual)
- DMFT index (decayed, missing and filled tooth)
- Clinical attachment loss (CAL) in mm (distance from the cementoenamel junction to the bottom of the pocket/sulcus)

Other parameters collected such as positive suppuration of total sites and tooth mobility grade from I to III were also collected for each patient.

For the collection of biofilm samples, the two deepest pocket sites in four quadrants were selected. To prevent contamination with saliva, cotton rolls were used for isolation. Two biofilm samples were collected by inserting a sterile curette into the gingival pocket, transferred to an Eppendorf tube, stored in transport medium with liquid nitrogen and then placed in a freezer at − 80 °C within less than 15 min until further processing.

The medication used to treat IBD was also recorded to analyze its influence on the expression of cytokines.

Moreover, the HBI (Harvey-Bradshaw Index) in CD patients and the pMS (partial Mayo Score) in UC patients were determined to classify disease activity as following (25–27):

HBI:

- Severe (17 +)
- Moderate (8–16)
- Mild (5–7)
- Remission (0–4)

pMS:

- Severe (8–9)
- Moderate (5–7)
- Mild (2–4)
- Remission (0–1)

### Laboratory examination

Total RNA was isolated from the biofilm samples, and the expression of cytokines and inflammatory mediators (IL-2, -6 and -10, metalloproteases (MMP)-7, -9 and CD-90) was determined using reverse transcription quantitative polymerase chain reaction (RT-qPCR). Moreover, integrin alpha L also called CD11a (ITGAL) was included as a positive control to demonstrate that inflammatory material was present in the biofilm samples.

RNA from the samples was extracted using the TRIzol™ method (Thermo Fischer Scientific, Waltham, Massachusetts, USA) according to the manufacturer’s protocol. The concentration and quality of the isolated RNA were determined using a NanoDrop photospectrometer (PeqLab, Erlangen, Germany). Next, the isolated RNA was transcribed into cDNA using a Quantitect Reverse Transcriptase kit (Qiagen, Venlo, Netherlands) according to the manufacturer’s protocol.

The cDNA was used to determine the expression of the markers by qPCR. For this analysis, specific QuantiTect Primer Assays (Qiagen, Venlo, Netherlands) were selected for the target genes, IL-2, IL-6, IL-10, MMP7, MMP9, and CD90, and a housekeeping gene, GAPDH (Table 1) (Qiagen, Venlo, Netherlands). SYBR Green Master Mix from Qiagen was used for subsequent qPCR according to the manufacturer’s instructions. qPCR was performed in a 7300 real-time PCR system (Thermo Fisher Scientific, Waltham, Massachusetts, USA). After 15 min of incubation in the instrument, 50 cycles were performed, with each cycle as follows: the first 15 s at 94° for the denaturation process, then 30 s at 55° for the annealing process, and 34 s at 72° for the extending process. Following qPCR, dissociation curve or melting curve analysis was performed for each plate, providing information on the homogeneity of the amplicons. All the samples were analyzed in duplicate, and the average CT was calculated. GAPDH served as an endogenous control. Only samples that showed a clear amplification curve of GAPDH and a CT value of less than 37 cycles were used for additional analyses. Samples that showed a higher ∆CT value for the endogenous control were not considered in further analyses, as it must be assumed that the quality of the obtained sample was insufficient. Samples with an acceptable CT value for GAPDH but no CT value for the target gene were considered undetected. For these samples, a mean CT value of 50 was assumed, which corresponded to the maximum number of cycles of qPCR in our analyses [25]. The ∆∆CT method was used to normalize the CT values using GAPDH as an endogenous control. The resulting ∆CT values were used for statistical analysis. A sample from the spleen was used as a positive control in all the PCR investigations.

**Table 1:** Product details and charge numbers of the used markers

Marker	Product name	Cat. No	Lot No
IL-2	Hs_IL2_1_SG	QT00015435	354,840,918
IL-6	Hs_IL6_1_SG	QT00083720	315,797,203
IL-10	Hs_IL10_1_SG	QT00041685	315,797,224
MMP7	Hs_MMP7_1_SG	QT00001456	315,797,201
MMP9	Hs_MMP9_1_SG	QT00040040	354,840,919
CD90	Hs_THY1_1_SG	QT00023569	354,840,947
CD11a	Hs_ITGAL_1_SG	QT00034006	361,111,662
GAPDH	Hs_GAPDH_1_SG	QT00079247	345,551,798

Higher ∆CT values indicate lower expression of the investigated target genes.

To show the relative change in expression rates, the fold change is used. A fold change of 2 means that the expression has doubled (28).

To minimize the possible bias of sampling from the sulcus, only one practitioner (J.F) took the samples. The term expression is used consecutively and describes the level of concentration of the mediators in the biofilm, since strictly speaking the expression is from cells from the gingival crevicular fluid (GCF) and this can only be sampled by methods other than ours.

The primary outcome of the study was the difference of the inflammatory markers in terms of their expression levels in the sulcus in each group. Secondary outcomes were the assessment of clinical parameters (plaque index, BOP, PD, DMFT, CAL).

### Statistical analysis

The study size was not statistically determined in advance, as we first wanted to establish the methodology (especially the laboratory examination) based on the included patients. Statistical analysis of the collected data was performed using SPSS, Version 25 software (IBM, Armonk, New York, USA). If not stated otherwise, data are presented as the mean ± standard deviation. For statistical comparison among the three groups, the non-parametric Kruskal–Wallis test was performed. Differences among groups were considered significant if the *p*-value was < 0.05, and if a statistically significant difference was detected, then we performed an additional pairwise comparison with a separate non-parametric Mann–Whitney *U*-test. For statistical comparison of the data (clinical parameters and expression) among the groups (either HC vs. CD or HC vs. UC), a non-parametric Mann–Whitney *U*-test with a significance level of *p* < 0.05 was performed. The fold change is calculated according to the 2^ − ΔΔCT formula for the significant values The Wilcoxon-Mann–Whitney *U*-test was performed to calculate the post-hoc power analysis.

## Results

### Study population

**Fig. 1:**
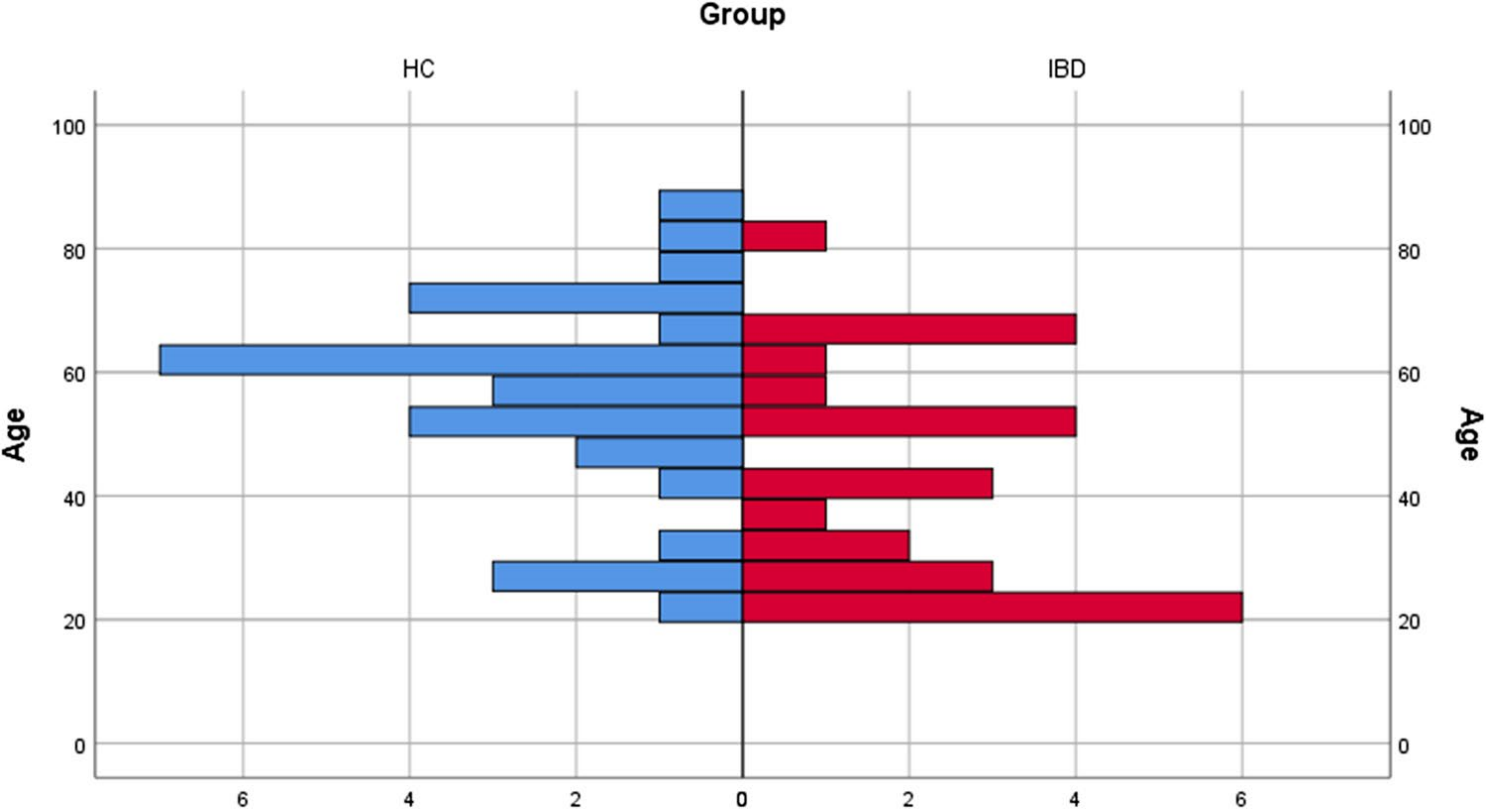
Showing the frequency of distribution between IBD and HC regarding the age

**Fig. 2:**
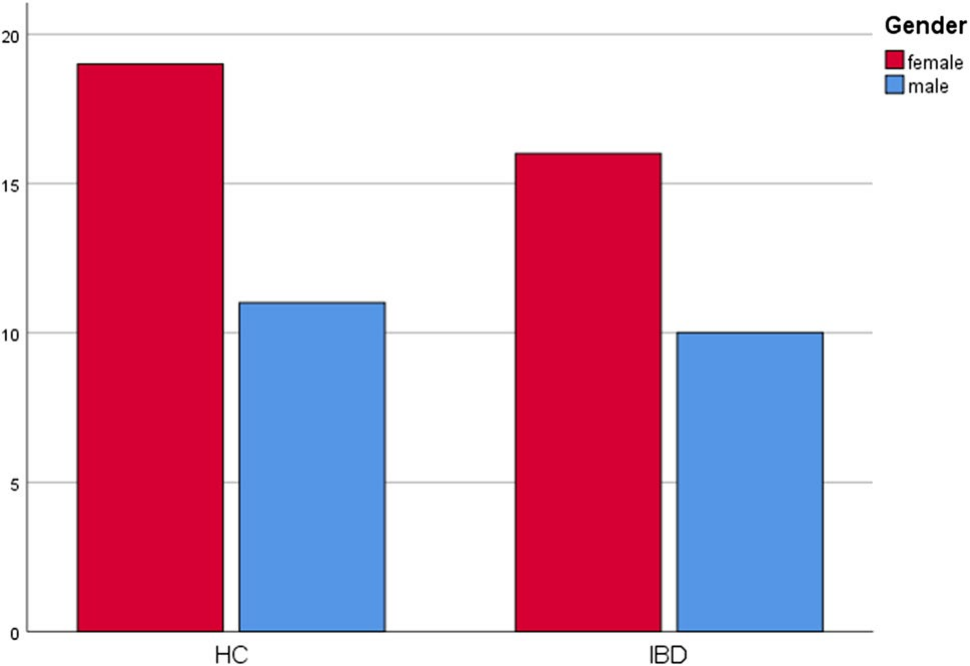
Showing the frequency of distribution between IBD and HC regarding the gender

A total of 26 IBD patients consisting of 16 females and 10 males were included in this study. Of the patients in the IBD group, a total of 7 (*n* = 2 male, *n* = 5 female) had UC. The mean age of these patients was 37 ± 14 years. There were 19 CD patients (*n* = 8 male, *n* = 11 female), with a mean age of 46 ± 14 years. The HC group consisted of 30 (*n* = 11 male, *n* = 19 female) patients with a mean age of 56 ± 13 years. The study groups were not matched in terms of age or gender. The frequency of distribution between IBD and HC regarding age and gender can be seen in Figs. 1 and 2 without statistic impact. None of the patients has shown a genetic background (neither for CD nor UC), but in one CD patient the family history was positive for colon cancer. All included patients were of Caucasian ethnicity. The year of initial diagnosis of IBD ranged from 1973 to march 2019 in our patients. Twenty patients in the IBD group were treated with a biological or immunosuppressive medication (*n* = 14 TNF inhibitors: *n* = 13 infliximab and *n* = 1 adalimumab; *n* = 8 other immunosuppressants: *n* = 6 vedolizumab and *n* = 2 ustekinumab), and 6 of them received no medication for IBD. Whereas the patient with the initial diagnosis in March 2019 received infliximab as primary therapy, *n* = 10 of the patients received glucocorticoids and others over the past years. In this regard, fistulas or exposed bone were not observed in the group of patients who received a TNF inhibitor medication.

There were no differences among the 3 groups regarding changes in the oral mucosa (the cheek, mucosa, tongue, vestibulum) or keratinized gingiva and no recessions or furcation was observed in any tooth. Moreover, there were no cases of dry mouth or noticeable salivation in the three groups.

### Clinical parameters

#### Mombelli Plaque Index (Mombelli PI)

The mean values of the Mombelli PI did not differ significantly between the HC and CD groups as shown in Tables 2 and 3. There was no trend between the study groups and the HC group, and the mean value in the HC group was grade 1.

**Table 2:** Mean values of clinical parameters (PSI, BOP, Mombelli Plaque Index) and ∆CT values of cytokines (IL-2, -6, -10), MMP-7 and -9, and CD90 in group 0 (healthy control HC) and group 1 (Crohn’s disease CD) patients with the corresponding *p*-value (significant level *p* < 0.05), test power, and fold change. With significance in BOP (*p* = 0.001), IL-10 (*p* = 0.004), and MMP-7 (*p* = 0.032)

	Group 0 (HC) *n* = 30	Group 1 (CD) *n* = 19	*p*-value	Test power	Fold change
∆CT IL-2	21.29 ± 4.73 (*n* = 29)	22.51 ± 4.12 (*n* = 15)	0.340	0.130	0
∆CT IL-6	15.35 ± 6.33 (*n* = 28)	12.91 ± 5.21 (*n* = 17)	0.215	0.136	5
∆CT IL-10	19.98 ± 5.76 (*n* = 28)	12.89 ± 8.06 (*n* = 18)	**0.004**	**0.892**	**136**
∆CT MMP7	12.36 ± 7.93 (*n* = 28)	7.23 ± 6.57 (*n* = 18)	**0.032**	**0.915**	**35**
∆CT MMP9	7.62 ± 4.02 (*n* = 26)	7.62 ± 5.57 (*n* = 17)	0.901	0.050	1
∆CT CD90	15.35 ± 6.57 (*n* = 29)	14.72 ± 7.52 (*n* = 14)	0.979	0.057	2
∆CT CD11a	9.95 ± 6.65 (*n* = 27)	7.53 ± 3.66 (*n* = 18)	0.660	0.292	5
Mombelli PI (grade 0–3)	1.17 ± 0.79 (*n* = 30)	1.53 ± 0.84 (*n* = 19)	0.178	0.301	-
BOP (in %)	11.75 ± 6.77 (*n* = 30)	30.91 ± 26.47 (*n* = 19)	**0.001**	**0.898**	**-**
PD (mm)	2.53 ± 0.76	2.71 ± 0.90	**0.000**	**0.108**	**-**
DMFT	15.57 ± 8.90	13.32 ± 9.76	0.447	0.123	-
Number of teeth	23.87 ± 6.57	24.32 ± 6.38	0.670	0.055	-
Suppuration total sites	0.13 ± 0.43	0.05 ± 0.23	0.545	0.117	-
Tooth mobility grade I	0.60 ± 1.77	0.26 ± 0.65	0.906	0.132	-
Tooth mobility grade II	0.00 ± 0.00	0.00 ± 0.00	1.000	0	-
Tooth mobility grade III	0.00 ± 0.00	0.11 ± 0.46	0.209	/	-
CAL ≥ 2 mm total sites	5.70 ± 6.46	2.79 ± 7.01	0.069	0.290	-

**Table 3:** Showing the mean values of clinical parameters (PSI, BOP, Mombelli Plaque Index) and ∆CT values of cytokines (IL-2, -6, -10), MMP-7 and -9, and CD90 in group 0 (healthy control HC) and group 2 (ulcerative colitis UC) patients with the corresponding *p*-value (significant level *p* < 0.05), test power, and fold change. With significance in IL-10 (*p* = 0.022)

	Group 0 (HC) *n* = 30	Group 2 (UC) *n* = 7	*p*-value	Test power	Fold change
∆CT IL-2	21.29 ± 4.73 (*n* = 29)	18.85 ± 6.03 (*n* = 6)	0.356	0.158	5
∆CT IL-6	15.35 ± 6.33 (*n* = 28)	18.37 ± 5.42 (*n* = 6)	0.318	0.190	0
∆CT IL-10	19.98 ± 5.76 (*n* = 28)	13.83 ± 5.99 (*n* = 7)	**0.022**	**0.650**	**71**
∆CT MMP7	12.36 ± 7.93 (*n* = 28)	12.03 ± 8.25 (*n* = 7)	0.825	0.050	1
∆CT MMP9	7.62 ± 4.02 (*n* = 26)	11.25 ± 6.72 (*n* = 7)	0.375	0.307	0
∆CT CD90	15.35 ± 6.57 (*n* = 29)	13.25 ± 6.63 (*n* = 7)	0.253	0.110	4
∆CT CD11a	9.95 ± 6.65 (*n* = 27)	11.58 ± 6.05 (*n* = 7)	0.379	0.088	0
Mombelli PI (grade 0–3)	1.17 ± 0.79 (*n* = 30)	1.00 ± 0.82 (*n* = 7)	0.635	0.076	-
BOP (in %)	11.75 ± 6.77 (*n* = 30)	18.17 ± 19.20 (*n* = 7)	0.894	0.172	-
PD (mm)	2.53 ± 0.76	2.51 ± 0.71	0.490	0.050	-
DMFT	15.57 ± 8.90	13.57 ± 9.83	0.482	0.077	-
Number of teeth	23.87 ± 6.57	27.57 ± 1.27	0.227	0.423	-
Suppuration total sites	0.13 ± 0.43	0.00 ± 0.00	0.690	/	-
Tooth mobility grade I	0.60 ± 1.77	0.00 ± 0.00	0.608	/	-
Tooth mobility grade II	0.00 ± 0.00	0.00 ± 0.00	1.000	0	-
Tooth mobility grade III	0.00 ± 0.00	0.00 ± 0.00	1.000	0	-
CAL ≥ 2 mm total sites	5.70 ± 6.46	4.14 ± 5.93	0.556	0.087	-

#### Bleeding on probing (BOP)

The mean value of BOP was significantly higher in the CD group than that in the HC group (*p* = 0.001, Table 2) whereas the mean BOP value did not differ significantly between the HC group and the UC group (Table 3). However, BOP tended to be higher in the UC group (mean value of 18.17%) than in the HC group (mean value of 11.75%).

#### Pocket depth (PD)

The mean value of PD was significantly higher in the CD group than that in the HC group as shown in Table 2 whereas the mean PD did not differ between the HC group and the UC group as shown in 3.

#### DMFT index

The mean value of DMFT index did not differ significantly between the HC and CD or UC groups (Tables 2 and 3). There was also no difference in relation to the total number of teeth between the groups.

#### Clinical attachment loss (CAL)

We evaluated the CAL values with at least ≥ 2 mm. The mean values of the CAL did not differ significantly between the groups (Tables 2 and 3).

#### Suppuration and tooth mobility grade from I to III

The mean values of suppuration did not differ significantly between the groups (Tables 2 and 3). There were no teeth with increased mobility grade II in the HC, CD or UC groups, or III between the HC and UC groups. And no significant differences of teeth with mobility grade III between the HC and CD groups. Moreover, the mean values of tooth mobility grade I did not differ significantly between the groups (Tables 2 and 3).

### Cytokine expressions

The expression level of CD-11a served as a positive control to demonstrate inflammatory material, and the expression of CD-11a in the three groups is shown in Tables 2, 3, 4, and 5.

**Table 4:** Showing the ∆CT values of cytokines (IL-2, -6, -10), MMP-7 and -9, and CD90 in patients without immunosuppressants compared to TNF inhibitors with the corresponding *p*-value (significant level *p* < 0.05), test power, and fold change. With significance in IL-10 (*p* = 0.007)

	No immunosuppressants	TNF-α inhibitors	*p*-value	Test power	Fold change
∆CT IL-2	21.38 ± 4.65 (*n* = 31)	20.43 ± 5.21 (*n* = 13)	0.748	0.086	2
∆CT IL-6	15.49 ± 6.32 (*n* = 32)	13.68 ± 5.29 (*n* = 11)	0.388	0.135	4
∆CT IL-10	19.52 ± 6.27 (*n* = 32)	13.04 ± 7.41 (*n* = 13)	**0.007**	**0.781**	**89**
∆CT MMP7	11.93 ± 7.88 (*n* = 32)	7.41 ± 6.86 (*n* = 13)	0.054	0.426	23
∆CT MMP9	8.11 ± 5.47 (*n* = 29)	8.66 ± 4.68 (*n* = 13)	0.391	0.060	1
∆CT CD90	15.43 ± 6.82 (*n* = 33)	15.29 ± 7.52 (*n* = 10)	0.796	0.050	1
∆CT CD11a	9.43 ± 6.35 (*n* = 31)	9.61 ± 5.40 (*n* = 13)	0.361	0.050	1

**Table 5:** Showing the ∆CT values of cytokines (IL-2, -6, -10), MMP-7 and -9, and CD90 in patients without immunosuppressants compared to immunosuppressants with the corresponding *p*-value (significant level *p* < 0.05), test power, and fold change. With significance in IL-10 (*p* = 0.014)

	No immunosuppressants	Other immunosuppressants	*p*-value	Test power	Fold change
∆CT IL-2	21.38 ± 4.65 (*n* = 31)	23.29 ± 4.40 (*n* = 6)	0.365	0.146	0
∆CT IL-6	15.49 ± 6.32 (*n* = 32)	14.16 ± 6.04 (*n* = 8)	0.636	0.081	3
∆CT IL-10	19.52 ± 6.27 (*n* = 32)	11.81 ± 6.98 (*n* = 8)	**0.014**	**0.798**	**209**
∆CT MMP7	11.93 ± 7.88 (*n* = 32)	10.30 ± 8.14 (*n* = 8)	0.521	0.077	3
∆CT MMP9	8.11 ± 5.47 (*n* = 29)	7.33 ± 4.46 (*n* = 8)	0.912	0.065	2
∆CT CD90	15.43 ± 6.82 (*n* = 33)	11.66 ± 4.94 (*n* = 7)	0.328	0.304	14
∆CT CD11a	9.43 ± 6.35 (*n* = 31)	7.33 ± 4.46 (*n* = 8)	0.912	0.150	4

**Fig. 3:**
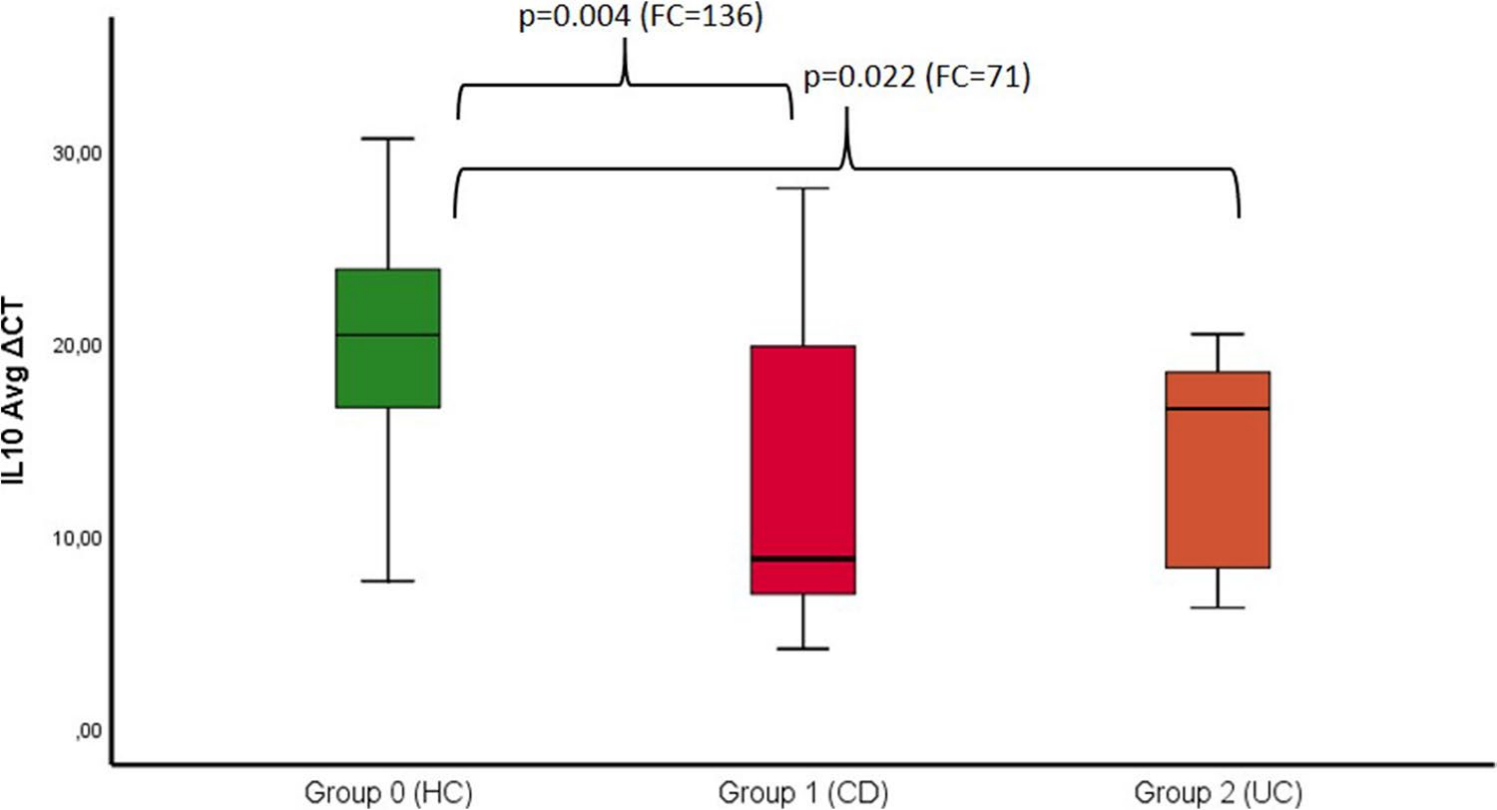
Showing the ∆CT of IL-10 in the three different groups (0 = HC vs. 1 = CD vs. 2 = UC) and fold change values with the corresponding *p*-value (significant level *p* < 0.05). With significance CD vs. HC (*p* = 0.004 and FC = 136) and UC vs. HC (*p* = 0.022 and FC = 71)

**Fig. 4:**
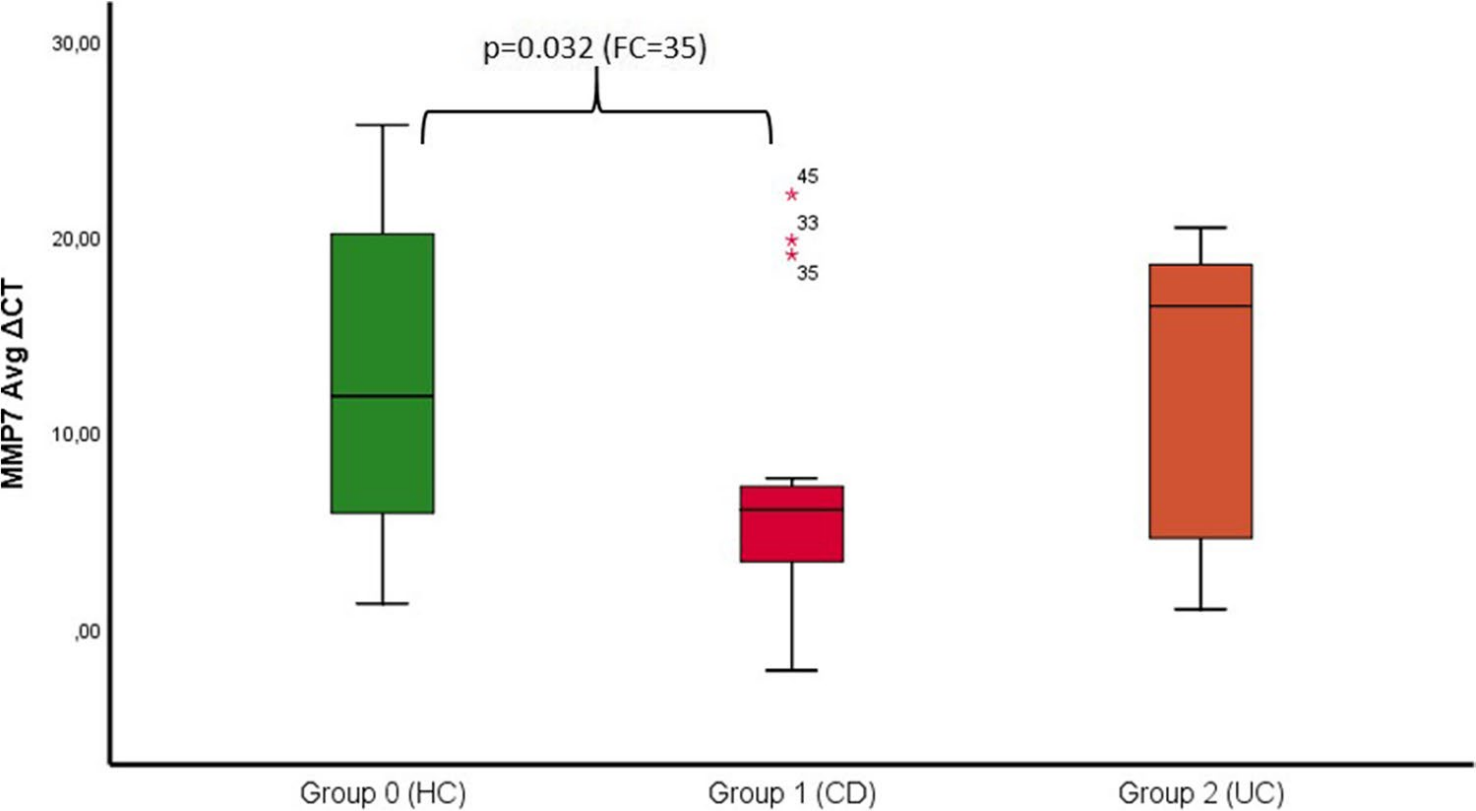
Showing the ∆CT of MMP-7 in the three different groups (0 = HC vs. 1 = CD vs. 2 = UC) and fold change values with the corresponding *p*-value (significant level *p* < 0.05). With significance CD vs. HC (*p* = 0.032 and FC = 35)

The expression of the anti-inflammatory cytokines IL-10 and MMP-7 was significantly higher in the CD group than that in the HC group (*p* IL-10 = 0.004 and *p* MMP-7 = 0.032), as shown in Table 2 and Figs. 3 and 4. However, no difference in MMP-9 was observed between the CD and UC groups compared to that in the HC group.

In the UC group, the expression of anti-inflammatory IL-10 was also significantly higher than that in the HC group (*p* = 0.022), as shown in Fig. 3 and Table 3.

The pro-inflammatory cytokines IL-2, IL-6, MMP9, and CD-90 showed no significant differences in expression in the CD and UC groups compared to that in the HC group (Tables 2 and 3).

**Fig. 5:**
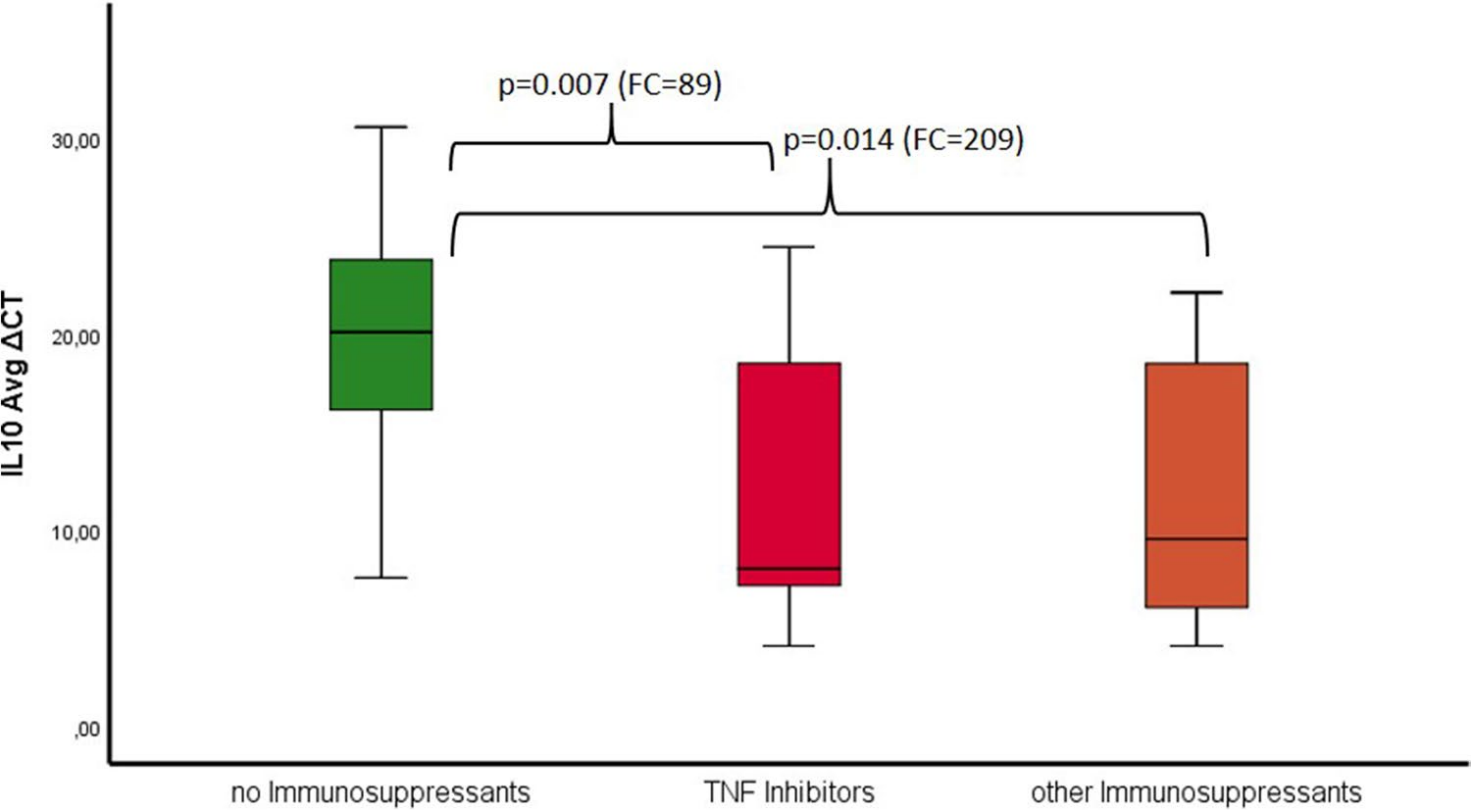
Showing the ∆CT of IL-10 in the three different groups (no immunosuppressants vs. TNF inhibitors vs. other immunosuppressants) and fold change values with the corresponding *p*-value (significant level *p* < 0.05). With significance no immunosuppressants vs. TNF inhibitors (*p* = 0.007 and FC = 89) and no immunosuppressants vs. other immunosuppressants (*p* = 0.014 and FC = 209)

Comparing the study groups in terms of the received medication, the expression of anti-inflammatory IL-10 was significantly higher in the group receiving TNF inhibitors than that in the group receiving no immunosuppressive medication, as shown in Table 4 and Fig. 5. The group that received immunosuppressants other than TNF-α inhibitors showed higher IL-10 expression than that of the group that did not receive immunosuppressants other than TNF inhibitors as shown in Table 5 and Fig. 5. Otherwise, for the other parameters, there were no differences among the groups regarding the different medications (Tables 4 and 5).

### Disease activity

The different disease scores of CD and UC patients and correlation to either clinical parameters or cytokine expressions are shown in Tables 6 and 7.

**Table 6:** Showing the ∆CT values of cytokines (IL-2, -6, -10), MMP-7 and -9, and CD90 in CD patients according to the disease activity and HBI scores

	CD remission (HBI)	CD mild (HBI)	CD moderate (HBI)	CD total group
∆CT IL-2	21.82 ± 4.66 (*n* = 8)	24.25 ± 2.73 (*n* = 5)	20.97 ± 1.17 (*n* = 2)	22.51 ± 4.12 (*n* = 15)
∆CT IL-6	12.24 ± 3.99 (*n* = 9)	11.52 ± 5.21 (*n* = 5)	**17.25 ± 5.34 (*n* = 3)**	12.91 ± 5.21 (*n* = 17)
∆CT IL-10	11.21 ± 6.48 (*n* = 10)	16.33 ± 9.84 (*n* = 5)	**15.70 ± 7.51 (*n* = 3)**	12.89 ± 8.06 (*n* = 18)
∆CT MMP7	5.74 ± 5.01 (*n* = 9)	5.74 ± 2.28 (*n* = 6)	**16.13 ± 6.91 (*n* = 3)**	7.23 ± 6.57 (*n* = 18)
∆CT MMP9	6.80 ± 1.10 (*n* = 9)	9.82 ± 8.47 (*n* = 6)	4.67 ± 0.98 (*n* = 2)	7.62 ± 5.57 (*n* = 17)
∆CT CD90	14.60 ± 7.00 (*n* = 7)	14.66 ± 8.35 (*n* = 5)	15.25 ± 4.55 (*n* = 2)	14.72 ± 7.52 (*n* = 14)
∆CT CD11a	9.60 ± 3.75 (*n* = 9)	6.13 ± 1.60 (*n* = 6)	4.12 ± 0.60 (*n* = 3)	7.53 ± 3.66 (*n* = 18)

**Table 7:** Showing the ∆CT values of cytokines (IL-2, -6, -10), MMP-7 and -9, and CD90 in UC patients according to the disease activity and pMS scores

	UC remission (pMS)	UC moderate (pMS)	UC total group
∆CT IL-2	18.98 ± 6.02 (*n* = 5)	18.24 (*n* = 1)	18.85 ± 6.03 (*n* = 6)
∆CT IL-6	18.34 ± 5.42 (*n* = 5)	18.52 (*n* = 1)	18.37 ± 5.42 (*n* = 6)
∆CT IL-10	13.04 ± 5.62 (*n* = 6)	**18.52 (*n* = 1)**	13.83 ± 5.99 (*n* = 7)
∆CT MMP7	10.95 ± 7.73 (*n* = 6)	**18.52 (*n* = 1)**	12.03 ± 8.25 (*n* = 7)
∆CT MMP9	10.04 ± 5.90 (*n* = 6)	**18.52 (*n* = 1)**	11.25 ± 6.72 (*n* = 7)
∆CT CD90	12.38 ± 6.21 (*n* = 6)	**18.52 (*n* = 1)**	13.25 ± 6.63 (*n* = 7)
∆CT CD11a	10.42 ± 5.21 (*n* = 6)	**18.52 (*n* = 1)**	11.58 ± 6.05 (*n* = 7)

Classification of patients according to their HBI disease activity scores showed that the patients included in our study all belonged to either the remission (HBI 0–4), mild (HBI 5–7), or moderate (HBI 8–16) class within the CD group (Table 6). The ∆CT values of the inflammatory markers in correlation to the disease activity are shown in Table 6. Compared to the ∆CT values of the whole CD group, no interpretation of the ∆CT values can be derived based on the small numbers of patients. Therefore, only the higher ∆CT values compared to the total group are marked. It can only be deduced that in the patients with a moderate disease activity (*n* = 3), an overexpression of IL-6, IL-10, and MMP-7 is seen.

Classification of patients according to their pMS disease activity score showed that two groups (remission pMS 0–1 and moderate pMS 5–7) were represented in our UC group (Table 7). The small number of patients does not allow any interpretation and the higher ∆CT values compared to the total group are marked. It can be seen here that there was an overexpression of IL-10, MMP-7, MMP-9, CD90, and CD11a only in the one patient belonging to the moderate group.

## Discussion

Periodontitis (P), which is characterized by the degradation of hard and soft tissues of the periodontium, is multifactorial, and the interplay among bacteria, inflammatory mediators, respective immune responses, and environmental factors (e.g., smoking) seems to be crucial (29). IBD is also characterized by an exaggerated immune response and the accompanying release of pro- or anti-inflammatory mediators. We know from the previous literature that some of the same cytokines are involved in IBD and P pathogenesis and that these cytokines are detectable outside the gut, namely, in the oral cavity (14–17). In this context, many studies have already described altered parameters in the periodontium, in particular, elevated periodontal parameters in IBD patients (4, 13, 30).

In a retrospective study, 229 IBD patients were compared with 135 healthy subjects regarding DPSI (Dutch PSI) and DMFT. While DMFT was significantly higher in IBD patients than in healthy subjects, DPSI did not differ between the groups regarding specific IBD medications (30). Other studies compared 99 CD patients and 80 UC patients with 74 periodontitis patients according to the DMFT, BOP, PD and plaque index parameters. Again, IBD patients had higher DMFT values than those of the other patients and CD patients had less BOP and lower plaque indices but deeper pocket depths than those of P patients (14, 31).

These findings agree partially with the clinical results of this study. Regarding CAL, DMFT and the Mombelli PI, no differences between IBD and healthy patients were observed. The BOP and PD mean value was significantly increased in CD patients compared to that in healthy patients, without any significance in UC patients. This may be due to the small number of UC patients included in the study. However, as P and IBD are both chronic inflammatory diseases, analyzing the expression of different cytokines in these diseases is crucial. In a previous study, gingival crevicular fluid was evaluated in IBD and chronic P patients with respect to IL-4, -6, -1ß, -18, and IFN-γ; no significant differences were found. Clinical parameters (BOP, CAL, and PI) were also not significantly different between the groups (17). In this study, samples were taken from the deepest pocket in IBD and healthy patients both non-periodontal diseased and were examined with respect to cytokine expression. Although IL-6 could be detected in inflamed gingiva tissues and in manifest periodontitis tissue by ELISA in a previous study (32) we could not detect any differences in IL-6 expression between healthy controls and IBD patients, even though the cytokine was detectable in all groups.

Regarding anti-inflammatory IL-10, we observed significant overexpression in both disease groups compared to the healthy group (CD *p* = 0.004, UC *p* = 0.022). IL-10 which serves to maintain intestinal homeostasis could therefore also be detected in biofilm in IBD patients. It is interesting to note that in the context of periodontitis, especially the aggressive form of periodontitis, the plasma concentration of IL-10 is lower than that in healthy patients (33, 34). The plasma concentration was not investigated in this study, but the overexpression of IL-10 in the IBD patients did not allow any conclusion to be drawn regarding the possible occurrence of periodontitis in IBD patients. However, the overexpression of IL-10 agrees with our clinical observation that the IBD patients had no signs of periodontitis.

Pro-inflammatory metalloproteases (MMPs), which are responsible for the breakdown of bone tissue and bone, were also assessed, since the expression of MMP-7 and MMP-9 could also be detected in the active inflammation phase of the intestine of IBD patients. Moreover, CD-90, which is known as a major inflammatory marker but not explicitly in IBD patients, was investigated in this study (35, 36).

However, a correlation between IBD and CD-90 expression in biofilm samples could not be established. Regarding MMP-9, we did not observe any significant differences. MMP-7 overexpression was only observed in the CD group in comparison to that in the HC group (*p* = 0.032), although as already mentioned, no clinical correlation was observed.

As known from the literature, the proportion of IL-2 cells is increased in patients with IBD compared to that in healthy people (37). In addition, inflamed gingiva shows a much higher infiltration of cells expressing IL-2 in contrast to healthy gingiva (38).

This finding does not correspond to our results within biofilm samples. There were no significant differences in the expression of IL-2 and correlation to clinical parameters between the IBD and healthy groups.

Another effect on the decreased expression of pro-inflammatory markers is reported in correlation of smoking. This was confirmed in a study of 25 smokers compared with 25 nonsmokers and examination of cytokine expression in plaque samples. Smokers showed significantly reduced TNF levels compared with nonsmokers (39). To eliminate this confounding effect on our groups as well as the control group, we excluded smokers within this study.

Considering the general role of the host response in P patients, medication influencing the immune reaction might be relevant. However, immunosuppressive therapy and its impact on the periodontal condition have already been evaluated and have shown reduced periodontal inflammation in immunosuppressed patients compared to that in non-immunosuppressed patients (40). In recent decades, monoclonal antibodies (e.g., infliximab IFX) have been developed and proven effective for the treatment of IBD (41–44). Even though a 30% rate of non-responders is described in the literature the success of the medication is indisputable (45). By neutralizing TNF, IFX induces apoptosis of Th-cells or pro-inflammatory cytokines (46, 47). In addition, IFX can regulate IL-10 production (48). This effect is reflected in this study. The overexpression of IL-10 was significant in the TNF inhibitor group compared to that in the group that did not receive TNF inhibitors (*p* = 0.007).

In another study, it was also shown that TNF inhibitors (IFX) significantly downregulated the infiltration of neutrophils in the inflamed intestinal mucosa and peripheral blood in CD patients. TNF inhibition thus markedly suppressed not only TNF but also the production of pro-inflammatory cytokines, such as IL-6, -IL-8, -1β, and MMPs (49, 50). This finding does not clearly agree with our results. In the TNF inhibitor group, the expression of MMP-7 was not significantly higher (*p* = 0.054) than that in healthy subjects. MMP-9 expression was approximately the same among the groups.

Moreover, other biological therapies showed no significant differences in this respect.

Considering these results and the described effects of TNF inhibitors, it was hypothesized that reduced clinical periodontal parameters would be observed. However, only BOP, although not significantly, was increased in the TNF inhibitor group compared to that in healthy individuals and those who received other immunosuppressants.

Larger numbers of patients and their disease activity and correlation to medication would be very interesting for further studies regarding the expression of inflammatory markers in the sulcus.

To analyze the exact relationship between the presence of periodontitis and IBD, the composition of the bacteria in the oral biofilm also needs to be determined. P disease is described as a dysbiotic inflammatory disease (12). Brito et al. found no significant differences in clinical parameters but an increased number of opportunistic pathogens in IBD patients (31). Further studies including patients with P disease and microbiological analyses are necessary.

There are a few shortcomings and limitations in this study that need to be mentioned and critically discussed. First, the small number of patients included in this study has to be mentioned; because the methodological approach had to be established, we did not include a larger number of patients for now. However, this small number of patients certainly reduced the evidence for the presence of differential expression among groups. Moreover, the lack of matching the patients from the study groups limits the validity of the results. Furthermore, this study did not determine the bacterial composition or include patients with a periodontal disease, which was why no link between IBD and periodontitis or differences in its severity could be proven by these study results, and further studies are necessary on this topic.

## Conclusion

We detected cytokine expression in plaque samples from IBD patients and found significant differences regarding the expression of IL-10 compared to that in healthy individuals. BOP and PD was significantly higher in CD patients, but no other significant differences in clinical periodontal parameters could be detected between healthy and IBD patients. Thus, we conclude that no clinical impairment of periodontal tissue occurs in IBD patients. Immunosuppressants of any kind led to overexpression of IL-10 in plaque samples from IBD patients. Further studies are needed to analyze cytokine expression in IBD and periodontitis patients to find similarities between these diseases and to provide an explanation for the clinical observation of the prevalence of periodontitis in IBD patients.
